# Tracheomediastinal Fistula: Rare Complication of Treatment with Bevacizumab

**DOI:** 10.7759/cureus.2419

**Published:** 2018-04-03

**Authors:** Rajat Thawani, Anna Thomas, Kshitij Thakur

**Affiliations:** 1 Internal Medicine Resident, Maimonides Medical Center; 2 Internal Medicine, Crozer-Chester Medical Center, Upland, USA

**Keywords:** bevacizumab, tracheomediastinal fistula, tracheal fistula, non-small cell lung cancer

## Abstract

Tracheomediastinal fistula is a rare condition caused by multiple etiologies. We present a case of a patient of lung carcinoma receiving chemotherapy. A 63-year-old woman presented to the emergency room with a two-month history of worsening cough and shortness of breath. She was being treated with pemetrexed and bevacizumab for Stage IV non-small cell lung carcinoma. Chest X-ray showed a mass in the lung with mediastinal adenopathy. Computed tomography (CT) scan showed a perforation, confirmed with bronchoscopy. She had a secondary infection and she was started on intravenous antibiotics. The patient decided to continue care in a hospice. We present a rare complication of bevacizumab which has been only reported once in literature. Bevacizumab is known to cause tracheal fistulas when coupled with like invasive procedures. In our case, the patient developed a fistula without any invasive interventions. We advise that physicians using bevacizumab should be aware of the possibility of having such fistulas.

## Introduction

Tracheomediastinal fistula is a rare condition usually with a fatal outcome [1]. A literature review done showed that the listed causes of this fistula include tracheal or mediastinal lymphoma [1], radiation/laser therapy [2,3], chemotherapy in patients with lung carcinomas, notably bevacizumab [4], and surgical interventions [5]. We present a case where a patient receiving chemotherapy with bevacizumab came to the emergency room (ER) with shortness of breath and worsening cough.

## Case presentation

A 63-year-old female presented to the emergency room with a two-month history of worsening cough and shortness of breath. Over the course of this time, her symptoms were worsening and her cough became non-productive. She was using 2L oxygen for her symptoms. Her past medical history included a history of Stage IV non-small cell lung carcinoma (adenocarcinoma) with adrenal metastasis. She received first-line chemotherapy with pemetrexed and carboplatin for three months to which she did not respond, after which she was switched to pemetrexed with biweekly bevacizumab. Her last chemotherapy was two weeks before presentation.

**Figure 1:**
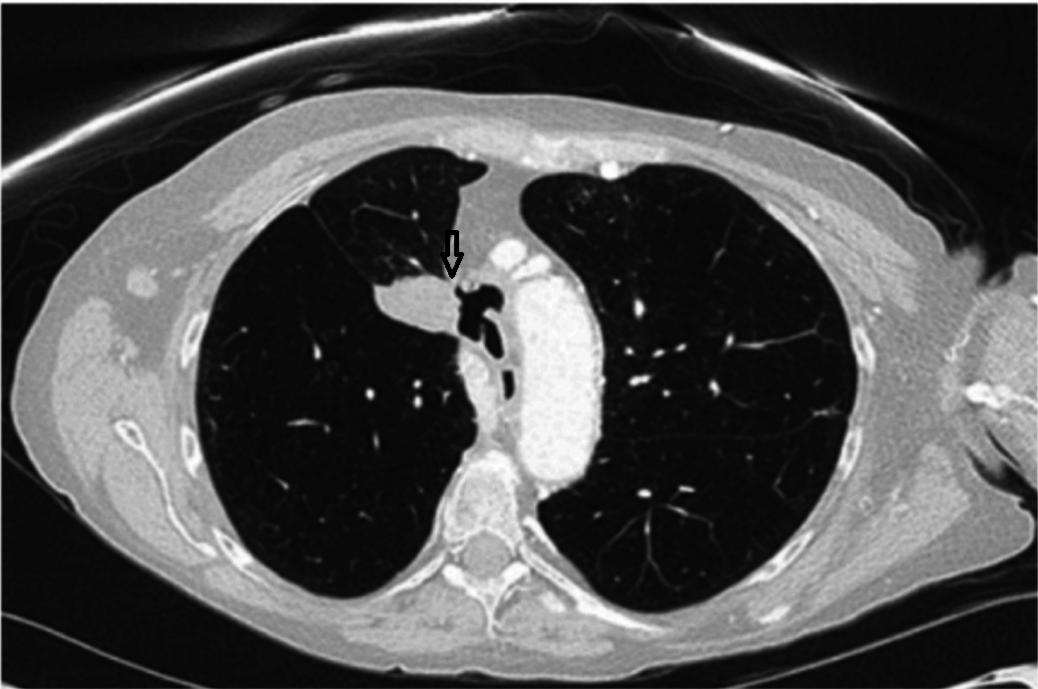
Computed tomography (CT) scan showing tumor near the mediastinum and formation of a fistula.

**Figure 2:**
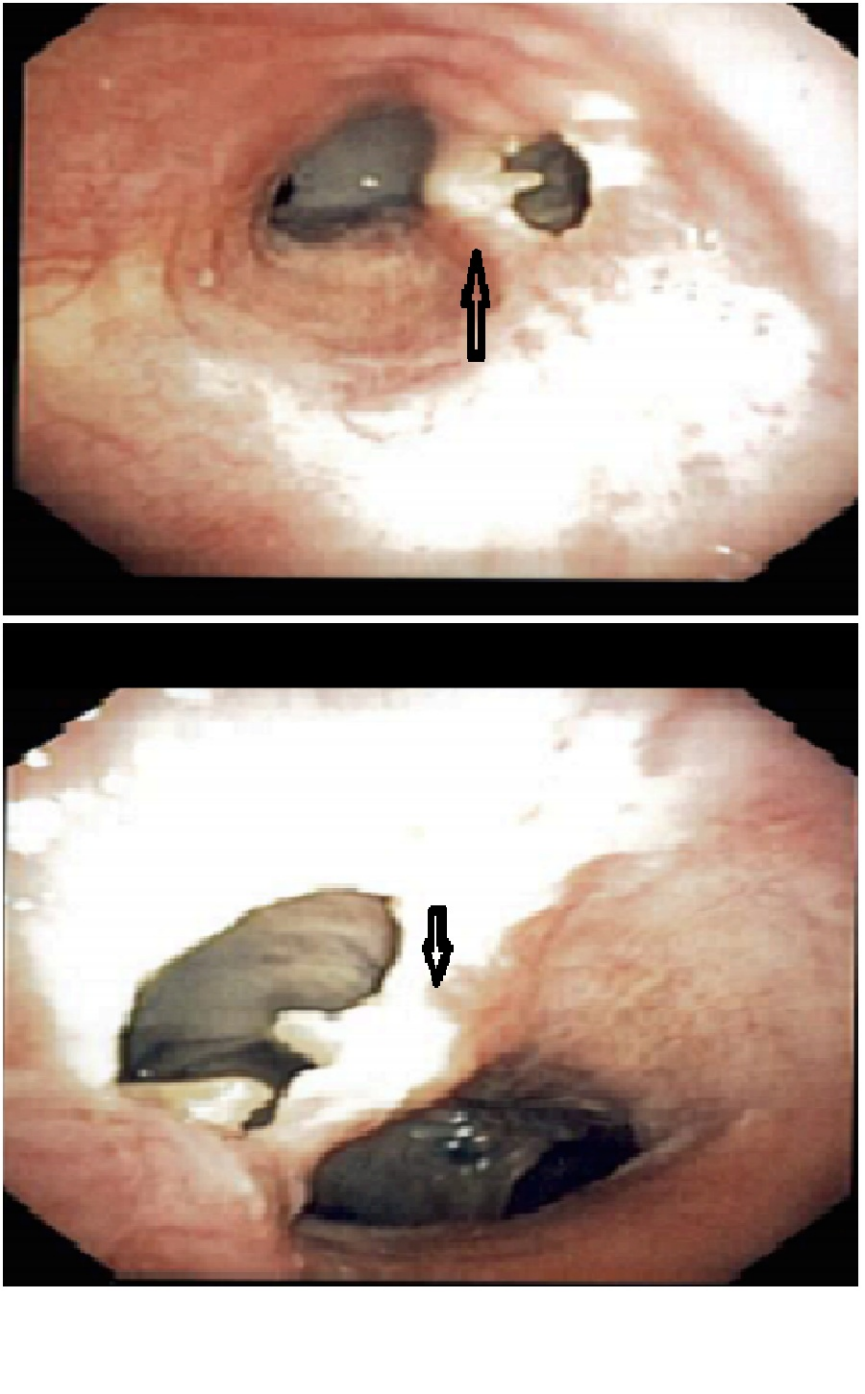
Bronchoscopy images showing the fistula. Arrow pointing at the fistula.

Significant vitals at presentation were the temperature of 99.6^o^ F, heart rate 102, respiratory rate 19, pulse oximetry 95% on 4L oxygen and blood pressure of 141/75 mm Hg. Examination showed bilateral equal and adequate air entry in lungs. Initial chest X-ray demonstrated a 2.5 x 4.5 x 3.9 cm upper lobe mass with mediastinal adenopathy and enlarged pulmonary arteries. Blood investigations showed lymphocytosis (white blood cells 14.2 x 10^9^/L). A computed tomography (CT) of her chest showed a possible tracheal invasion of the tumor with perforation (Figure 1). She was admitted and scheduled for bronchoscopy, which revealed a right-sided tracheomediastinal fistula (TMF) proximal to the carina (Figure 2). Blood cultures grew Streptococcus pneumonie and Klebsiella pneumonie. Thoracic surgery consult was obtained but surgery was deferred until her infection resolved. She was started on intravenous Vancomycin and Piperacillin-Tazobactam, and received them for 42 days with close follow-up. The patient wished against undergoing surgery later and decided to continue care in a hospice.

## Discussion

Bevacizumab has been shown to improve outcomes in patients with non-small cell lung cancer (NSCLC), apart from other malignancies like colorectal carcinoma, renal cell carcinoma, glioblastoma multiforme and gynecologic malignancies. At the same time, it has been associated with significant side effects like diarrhea, arterial and venous thromboembolism, hemorrhage and gastrointestinal perforations [6]. In patients of NSCLC, tracheoesophageal fistula (TEF) has been reported when bevacizumab is combined with radiotherapy [7]. There are multiple hypotheses about the mechanism of the causes of fistula. In NSCLC, the anti-angiogenic action of the drug in combination with esophageal or tracheal injury has been postulated as a cause of TEF. It is now a recognized adverse effect of patients who have received bevacizumab along with a multi-modality treatment of lung cancers.

On the other hand, TMF is an even rarer of bevacizumab reported only once by McCarthy and Hamel where a patient suffering from NSCLC received chemotherapy and radiation therapy [4]. But our patient never received any local treatment for the lung cancer, or never underwent any instrumentation and went on to develop TMF secondary to only bevacizumab.

There is no consensus on the treatment of this condition and is individualized. It may vary from surgical or endoscopic closure [2] to plasma coagulation [3], fibrin glue [5], or even autologous adipose-derived stem cells [2]. We treated our patient’s secondary infections before proceeding with definitive surgery as it is recommended in TEF [8-10].

The presentation of TMF in McCarthy and Hamel and our case was non-specific, and may not be detected by routine chest X-rays. We hypothesized that the TMF was formed secondary to treatment because there was no evidence of progression of the disease, and the patient was on treatment during the episode. Hence, we advise that physicians using bevacizumab should be aware of the possibility of having such rare fistulas and should consider it as an important differential in patients being treated with it. Any common or non-specific symptoms may be a sign of these rare fistulas and a thorough workup is warranted. Additionally, our case also shows that bevacizumab can cause these symptoms even in the absence of local therapy (radiation or surgery), unlike what has been published in the previous literature.

## Conclusions

Tracheomediastinal fistula could be a potentially lethal complication in patients being treated with bevacizumab. Physicians treating patients with this should be aware of this complication, and if symptoms suggest, terminate therapy early and take measures to treat the condition.
